# Fixation of tibial plateau fracture – risk factors for developing infection: a narrative review

**DOI:** 10.1530/EOR-24-0058

**Published:** 2024-12-02

**Authors:** Nicolas Franulic, Jose Tomas Muñoz, Tomas Pineda, Jose Laso, Rodrigo Olivieri, Steffen Schröter

**Affiliations:** 1 Knee Unit, Orthopedic Department, Hospital del Trabajador ACHS, Santiago, Chile; 2 Knee Unit, Orthopedic Department, Hospital Militar de Santiago, Chile; 3 Universidad de los Andes, Santiago, Chile; 4 Knee Unit, Orthopedic Department, Hospital el Carmen, Santiago, Chile; 5 Knee Unit, Orthopedic Department, Hospital Barros Luco Trudeau, Santiago, Chile; 6 Universidad Andrés Bello, Facultad de Medicina. Santiago, Chile; 7 Department of Traumatology and Reconstructive Surgery, Diakonie Klinikum GmbH Jung-Stilling-Krankenhaus, Siegen, Germany

**Keywords:** knee, fracture-related infection, tibial plateau fractures

## Abstract

- Fracture-related infection (FRI) after tibial plateau open reduction and fixation is a common complication that leads to catastrophic sequelae and substantial economic costs, making prevention paramount.
- To facilitate an appropriate approach, it is useful to classify risk factors based on patient-related factors, injury-related factors, and management-related factors.
- Patient-related factors like smoking have a great amount of evidence establishing their relation with FRI. Diabetes and obesity might be associated, but evidence is somewhat conflicting. Nevertheless, smoking cessation and a multidisciplinary approach for these pathologies are essential to prevent FRI.
- Injury-related factors like high-energy fractures and acute compartment syndrome have compelling evidence relating them to FRI and must be acknowledged as inherent factors. While the exposure of the fracture has been associated with infection, open fractures are yet to be confirmed as directly related to FRI in tibial plateau fractures. Likewise, early antibiotic prophylaxis and surgical debridement are mandatory.
- As for management-related factors, increased surgical time emerges as a strong predictor for FRI. Evidence regarding the number of surgical approaches and plates shows a trend toward an increase in FRI prevalence. With respect to external fixator installation and removal, pin-plate overlapping is yet to be confirmed or ruled out as risk factors.

## Introduction

The incidence of fracture-related infection (FRI) in the lower limb reaches a rate of 23−30% and 2−23% (1), specifically in the case of tibial plateau fractures (2, 3).

While previously reported rates of deep infection following open reduction and fixation of tibial plateau fractures were as high as 80% (4, 5), thanks to the introduction of new approaches with reduced periosteal detachment (6, 7, 8), new implants, and sequential two-stage management involving initial external fixation followed by definitive internal fixation (1, 9), infection rates have decreased. However, their incidence in the literature remains significant, ranging between 1% and 6% in the case of closed tibial plateau fractures and up to 30% in open fractures (10, 11). Unfortunately, this complication leads to sequelae such as chronic pain, post-traumatic arthritis, deformity, loss of function, and substantial economic costs for healthcare systems, making prevention paramount in the pursuit of successful treatment (12, 13).

The above has led different groups to try to identify the main risk factors for the occurrence of FRI in patients undergoing reduction and fixation of tibial plateau fractures, giving rise to heterogeneous literature and a multiplicity of elements that may lead to infection.

The objective of this article is to present the current literature regarding the most relevant predictive factors for FRI in tibial plateau fractures treated by open reduction and fixation in order to provide the reader with a clear understanding of the factors related to the patient (smoking, obesity, and diabetes mellitus), injury (high-energy fracture, open fracture, and concomitant compartment syndrome), and treatment (use of external fixator, increased surgical time, quantity of plates, and number of surgical approaches). Risk factors mentioned in the literature, such as the variation of the approach in the form of a ‘lazy S’ (14), use of bone allograft (11, 15), or the use of arthroscopy in high-energy tibial plateau fractures, will not be described in detail due to both limited evidence and the recent emergence of evidence that dismisses their increased risk of developing FRI (15, 16, 17, 18).

## Smoking

Smoking is a well-established risk factor associated with impaired healing, delayed union, nonunion, wound-healing failure, and infections (19, 20). Despite the widely accepted role of postoperative smoking cessation in reducing complications, its impact on infection rates following the surgical treatment of tibial plateau fractures remains uncertain (20, 21).

In a multicenter retrospective study, Chan *et al.* failed to demonstrate a definitive association between smoking and postoperative infection risk (22). In contrast, Zhu *et al.* reported in a prospective study a notable odds ratio (OR) of 5.68 for developing surgical site infections (SSIs) among smokers (23). When considering bicondylar tibial plateau fractures, Morris *et al.*, in a retrospective study, found that smoking increased the likelihood of reoperation due to deep infection by 2.4 times, highlighting it as the sole patient-modifiable predictor of deep infection in this series (7).

Olszewski *et al.* reported a statistically higher infection rate in their univariate analysis, but this significance did not persist in their multivariate analysis (24). Similarly, Parkkinen *et al.* identified tobacco use as a significant risk factor in their univariate analysis but failed to confirm its independent risk status in the multivariable analysis (25). Conversely, Ruffolo *et al.* did not detect a statistically significant association between tobacco use and infection or nonunion rates in their study of 138 adult patients surgically treated for bicondylar tibial plateau fractures (8). More recently, Li *et al.*, in a retrospective study involving 398 patients with tibial plateau fractures, reported an OR of 4.79 for SSI associated with tobacco consumption (11). Furthermore, in a comprehensive systematic review involving all tibial plateau fractures, Shao *et al.* identified an OR of 2.13 for SSI (1).

Variations in study outcomes may partially stem from differences in reporting smoking intensity and criteria used to determine the presence of infection. Despite the conflicting evidence and inconsistent results demonstrated in some studies, it is widely accepted that patients should be counseled against smoking after injury because it is a self-modifiable risk factor.

## Obesity and diabetes

The role of obesity and diabetes as potential risk factors for postoperative infections in tibial plateau fractures has been studied, yielding diverse and inconsistent outcomes in the literature.

In a retrospective cohort of 132 patients with high-energy tibial plateau fractures, Ruffolo *et al.* reported that a body mass index (BMI) higher than 35 did not result in an increased likelihood of reoperation due to deep infection (8). In contrast, Ma *et al.,* in a retrospective study of 676 patients treated with open reduction and internal fixation (ORIF) for closed tibial plateau fractures, reported an OR of 1.58 associated with a BMI over 26 (26). Notably, Henkelman *et al.*, in one of the largest published series, identified BMI as an independent risk factor correlated with an elevated risk of FRI (27).

The relationship between diabetes and postoperative infections remains uncertain. In a retrospective cohort of 302 patients treated for high-energy tibial plateau fractures, Morris *et al.* did not observe a significant association between diabetes and the risk of reoperation for deep infection (7). Furthermore, Chan *et al.*, in a retrospective study of 210 consecutive operatively treated patients, did not conclusively establish a relationship between diabetes and postoperative infection (22). Conversely, Basques *et al.* reported an adjusted relative risk (RR) of 1.6 for diabetes in a multivariable analysis involving 59 tibial plateau fractures (28). In a comprehensive analysis of infection risk factors after ORIF for high-energy tibial plateau fractures, Olszewski *et al.* identified diabetes and alcohol abuse as the only patient factors linked to infection, with ORs of 3.2 and 1.7, respectively (24).

## High energy

High-energy tibial plateau fractures are severe injuries associated with a high rate of complications and have been recognized as one of the main risk factors for FRI (9, 27, 28).

In a multivariate analysis involving 59 tibial plateau fractures, Basques *et al.* emphasized the significance of high-energy tibial plateau fractures, reporting an RR of 2.7 for these fractures (28).

High-energy fractures are often associated with other established risk factors, such as open fractures or acute compartment syndrome (ACS), that may also be considered a consequence of the initial traumatic event. These factors include extensive soft-tissue damage, increased fracture complexity, open fractures, compartment syndrome, extended surgical durations, prolonged hospitalization, the use of external fixation, and the necessity for multiple surgical approaches and plates for fixation (8). The complexity of these fractures makes it difficult to identify their individual impact.

An elevated RR of infection has been established when categorizing Schatzker IV–VI or OTA/AO Type C fractures as high-energy injuries. Recently, in a multicentric retrospective cohort study involving 2106 surgically treated tibial plateau fractures, Henkelman *et al.* reported a significantly higher FRI rate in Type C (OTA/AO Classification) fractures in comparison to B fractures (27).

Moreover, Momaya *et al.* (9) and Li *et al.* (11) have shown that Schatzker V–VI fracture patterns independently contribute to infection risk following surgical treatment in adults. These findings were confirmed by other working groups (8, 29, 30). Nevertheless, subanalysis indicates that patients over the age of 65 with Schatzker IV–VI tibial plateau fractures do not exhibit a heightened infection risk. (9) The variation in infection risk profiles between the two age groups can be attributed to the substantial energy involved in accidents among the younger population (4, 31, 32, 33, 34). This observation suggests that fracture classification may not accurately reflect the true severity of injury in the older population.

While some risk factors may lie beyond the direct control of healthcare providers or are influenced by challenging-to-manage variables. It seems critical to recognize high-energy mechanisms as an inherent and main factor. Therefore, perioperative strategies to mitigate associated infection risks should be considered in every case.

## Open fracture

The exposure of a fracture generally involves wound contamination and greater damage to soft tissues. It may be argued that this is the most important predictive factor related to FRI of tibial plateaus reported in the literature (1, 3, 7, 8, 9, 15, 24, 35, 36).

In their retrospective series of patients with bicondylar fractures, Morris *et al.* observed an infection rate of 14.2% (43/302 patients), which indicates that patients with open fractures had a 3.4 times higher risk of developing a deep infection (OR = 3.44; *P* = 0.003) (7). In 2013, Colman´s group described, after a multivariate logistic regression analysis, that fracture exposure is an independent predictor of infection (OR = 7.02; *P* < 0.001), highlighting that a higher degree of open fracture – according to the Gustilo and Anderson classification – corresponds to a higher risk of infection (grade I: 14.3%, grade II: 40%, grade III: 50%; *P* < 0.0001) (3).

Furthermore, a meta-analysis published in 2017 (1) included eight studies with 2214 tibial plateau fractures treated by ORIF, resulting in 219 cases of FRI. Fracture exposure was identified as the main risk factor with an OR of 3.78. In the same year, the results of a multicenter prospective study involving 235 patients were published. After multivariate analysis, only open fracture (OR = 3.31) and active tobacco use (OR = 5.68) were significant predictive factors of infection (23).

Without going any further, recently Monllau *et al.* also observed in their series of 124 patients that fracture exposure is an independent risk factor for infection, describing that out of their 13 treated exposed tibial plateau fractures, five developed FRI (*P* = 0.002) (15).

Despite the aforementioned, a multicenter study published by Olszewski *et al.* (24) described an incidence of 13% in 123 patients with open tibial plateau fractures, with their multivariate analysis ruling out exposure as a predictor of infection. Regarding Gustillo classification, the average time elapsed from the accident to coverage for IIIB and IIIC fractures was 14.06 days. Within the first 4 months, 6.7% of grade I, 13.7% of grade II, 65% of grade IIIA, 24% of grade IIIB, and 25% of grade IIIC developed an infection.

Regarding coverage, a recent retrospective cohort study of patients with type IIIB and C open tibial plateau fractures showed that late flap coverage (>7 days) versus early coverage had significantly higher rates of deep infection (83.3% vs 16.7%) (*P* = 0.02) and reintervention (71.4% vs 33.3%) (*P* = 0.08) (37).

## Compartment syndrome

The association of ACS and tibial plateau fractures is well known due to their usual high-energy mechanism and the resulting soft tissue damage, with an estimated incidence between 4.3% and 27% (38, 39). However, the correlation of ACS as a risk factor in the development of an FRI in tibial plateau fractures remains contro­versial (39).

Initial studies state that the presence of ACS and four-compartment fasciotomies were not correlated with a higher incidence of FRI. In 2010, Hak *et al.* reported no deep infections in 12 patients with tibial plateau fractures and ACS treated with open fasciotomies, in comparison to a 6.25% of deep infection rate in the patients without this diagnosis (40). Similarly, Ruffolo *et al.*, in their retrospective review of 140 bicondylar tibial plateau fractures, reported a 23.6% rate of FRI, concluding that ACS had no statistically significant association with the infection rate (8). More recently, Thabet *et al.* reported only one deep infection (5.3%) in tibial plateau fractures with an ACS diagnosis, affirming that fasciotomies do not increase the risk of developing FRI (41).

On the contrary, numerous authors have reported an association between ACS and FRI (1, 7, 9, 25, 27, 39, 42, 43). In 2013, Morris *et al.* reported an OR of 3.81 for the development of FRI in tibial plateau fractures with concomitant ACS after multivariable logistic regression. The group of Momaya *et al.* described an OR of 3.35 and 2.38 for early and late infection, respectively (9). Blair *et al.* and Parkkinen *et al.* reported similar results, with an OR of 14.70 and 4.5 for ACS and four-compartment fasciotomy, respectively (25, 43). Likewise, Dubina *et al.* described a higher rate of infection with an OR of 7.27 (44).

To our knowledge, the study published by Shao *et al.* (1) is the only systematic review with meta-analysis on this subject. They included 2214 tibial plateau fractures and described a 9.9% incidence of SSI. An OR of 3.53 was reported in relation to infection risk in tibial plateau fractures with ACS.

Other related and controversial topics are the timing of fasciotomy closure, fasciotomy technique (single vs dual incision) and the correlation with the development of deep infection (38). Zura *et al.* (45) published their results of 81 fractures with associated ACS, with 23% of them developing deep infections. Nonetheless, they found no correlation between the diagnosis of infection and the time of fasciotomy closure. Conversely, in the same study mentioned above, Rufolo *et al.* reported a deep infection OR of 7.5 for patients with fasciotomies closed during or after definitive surgery, with a deep infection rate of 50% (8). Dubina *et al.* reported a 7% infection risk increase per day if the fasciotomy closure was delayed (44). In a recent multicentric study published by Henkelmann *et al.*, these results were found to be consistent (27). A more recent study, published by the group of Dubina *et al.*, found that simultaneous ORIF with fasciotomy closure was more likely to reduce FRI (44). Regarding fasciotomy technique, it appears that it has no impact on FRI rate (38).

## External fixator

**Figure 1:**
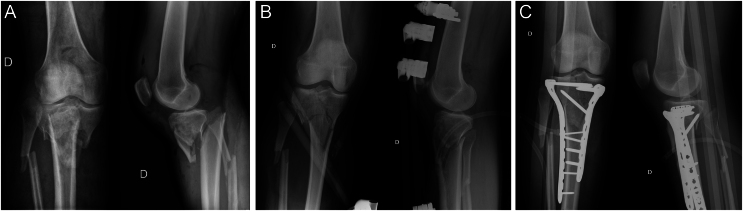
The strategy of performing a two-stage surgery can reduce the risk of infection in tibial plateau fractures with extensive soft tissue involvement, exposed fractures, or in the presence of acute compartment syndrome. (A) Tibial plateau fracture – Schatzker VI with severe displacement and concomitant acute compartment syndrome. (B) Temporary reduction and fixation with external fixator after fasciotomies of the four compartments of the leg. (C) Reduction and definitive osteosynthesis with two plates.

The treatment of high-energy tibial plateau fractures sometimes requires a two-stage surgery – due to Schatzker type IV–VI, ACS, or open fractures – requiring first an external fixator (ExFix) to allow the healing of the soft tissue envelope to perform ORIF in a second surgery (Fig. 1). With this premise, some questions arise regarding the risks and benefits of this conduct and the correlation of tibial plateau fractures surgically intervened with an ExFix and an increase of FRI.

Initial reports, such as the one published by Egol *et al.*, reported a low incidence of deep infection in their series of temporary spanning ExFix for tibial plateau fractures (46). Likewise, others have reported no statistical difference in deep infection rate or association with FRI for spanning ExFix in tibial plateau fractures (9, 47). The same was reported by Morris *et al.* in their retrospective study including 302 patients, finding no association between spanning ExFix and deep infection in their multivariate analysis (7). Three systematic reviews have reported no significant differences in overall infection rates and only found an association between higher superficial infection rates and fixation with ExFix (48, 49).

In contrast, other authors like Parkinnen *et al.* and Olszewski *et al.* described an OR of 3.9 and 2.07 as a risk factor in their multivariable regression analysis, respectively (24, 25). The same was reported by the group of Coelho *et al. a*nd Monllau *et al.*, with an infection rate of 57.1%. They also stated an association of higher infection risk in the ExFix patient group (15). Systematic reviews published by Metcalfe *et al.* and Zhao *et al.* comparing ExFix versus ORIF reported a significant correlation between the ExFix group and the development of any type of infection – superficial or deep (50, 51). Furthermore, in their systematic review aimed specifically to identify and quantify risk factors for SSI in tibial plateau fractures treated with ORIF, Shao *et al.* reported an OR of 2.07 for ExFix, only after excluding one study due to high heterogeneity (1).

In 2012, Laible *et al.* reported no correlation between infection and pin-plate overlapping (52). The same was stated by Ruffolo *et al.* in 2016 (8). Conversely, Shah *et al.* described a higher risk of infection for overlapping placement of ORIF for tibial plateau and pilon fractures (53). A recent retrospective study by Haase *et al.,* including 244 patients, reported a 27.7% infection rate and an RR 3.01 for the overlapping group (54).

Concerning the removal of the ExFix before the surgery, Stenquist *et al.* reported no statistical difference in deep infection rates between completely removing or retaining the ExFix, or between prepping the entire ExFix versus only the pins (55). The same results were published recently by Moon *et al.*, stating no correlation between the removal or prepping and maintenance of the ExFix (56).

## Increased operative time

The definitive surgical resolution of tibial plateau fractures requires advanced reduction skills and careful soft tissue management, possibly resulting in prolonged operative time. In relation to this, many studies have analyzed the relationship between prolonged surgical time and infection.

In their case–control study of 309 patients who underwent ORIF for tibial plateau fractures, the group of Colman (3) reported 24 cases of infection. They evaluated operative time, among other variables, as risk factors for the development of infection. Their multivariate logistic regression analysis concluded that longer operative times are an independent predictor of postoperative SSI in tibial plateau fractures (OR = 1.78; *P* = 0.01), with a cut-off value of 2.8 vs 2.2 h when comparing infected vs non-infected patients. In their discussion, they declare that their results suggest that for every extra hour of operative time, the risk of a postoperative infection increases by approximately 78%.

Lin *et al.* (42) reported a contemporary retrospective study including 256 cases. They found a total of 20 SSIs (7.8%) and identified prolonged surgical time as an independent risk factor (OR = 2.7; *P* < 0.001). These results were confirmed by Li *et al.* (11) in their retrospective analysis of 370 tibial plateau fractures. They found a total of 21 SSIs and concluded that prolonged surgical times showed a higher risk of infection (OR = 2.72; *P* = 0.020). Zhu *et al.* (23) performed an observational study with prospectively collected data, including 742 patients, of whom 20 patients (2.7%) developed SSI. Increased surgical time was associated with an increased OR for infection development (OR = 4.998; *P* = 0.038).

Furthermore, in 2018, the group of Ma *et al.* published a retrospective study of patients with closed tibial plateau fractures treated with ORIF, in which they described that a surgical time longer than 138 minutes is an independent risk factor for deep infection after performing their multivariate logistic analysis (26). Similarly, the group of Coelho *et al.* (15) found that a total surgical time above 158 min could increase the risk of infection; however, this variable did not reach statistical significance in their study (*P* = 0.07).

Although all the aforementioned authors recognized the influence of trauma severity, patient-related characteristics, difficult reduction, and extensive soft tissue stripping on the operative time, and emphasized the importance of thorough surgical planning and having an experienced surgical team, only Zhu *et al.* (23) discuss the possibility of closed reduction and minimally invasive osteosynthesis on the already severely damaged tissue. This approach, or limiting the number of surgical approaches to decrease surgical time, remains controversial and should be investigated with high-quality literature.

## Number of surgical plates and approaches

An often less considered point is the number of plates and surgical approaches used in the treatment of tibial plateau fractures and its relation with the incidence of FRI. The first one may imply more complex fracture patterns requiring several stable constructs to achieve anatomical reduction and stable fixation. The second, on the other hand, may lead to further periosteal stripping, bone devascularization, and ultimately higher risk of infection depending on the number of fragments and operative time.

Morris *et al.* in their retrospective study found a higher risk of infection in patients treated with both dual plating and double incision (OR = 3.19), adding that even though this may be a modifiable variable, the strategy was mandatory because of the unstable pattern of the fracture and a reflection of the injury severity (7). Olszewski *et al.* had similar findings in their retrospective study of 1287 cases, with an OR = 1.73 for dual plating (24). The authors comment on the importance of the injury’s energy and emphasize that some technical skills may be an issue when reducing the risk of infection. A recent retrospective case–control study by Olivieri *et al.* reported the use of two or more plates as an independent risk factor for infection (OR = 5.04) (57). In a similar fashion, in a novel retrospective study, Moon *et al.* (58) explored the role of plate locations and their role in the development of an infection, concluding that the addition of more occupied quadrants increases the incidence of infection: one quadrant with 8% of infection, two with 13%, three with 27.3%, and four with 100%. Although the number of plates was not associated with an increased risk of infection in their multivariate analysis, these results support the fact that further periosteal stripping is needed in order to install plates in more quadrants.

## Authors approach

**Figure 2:**
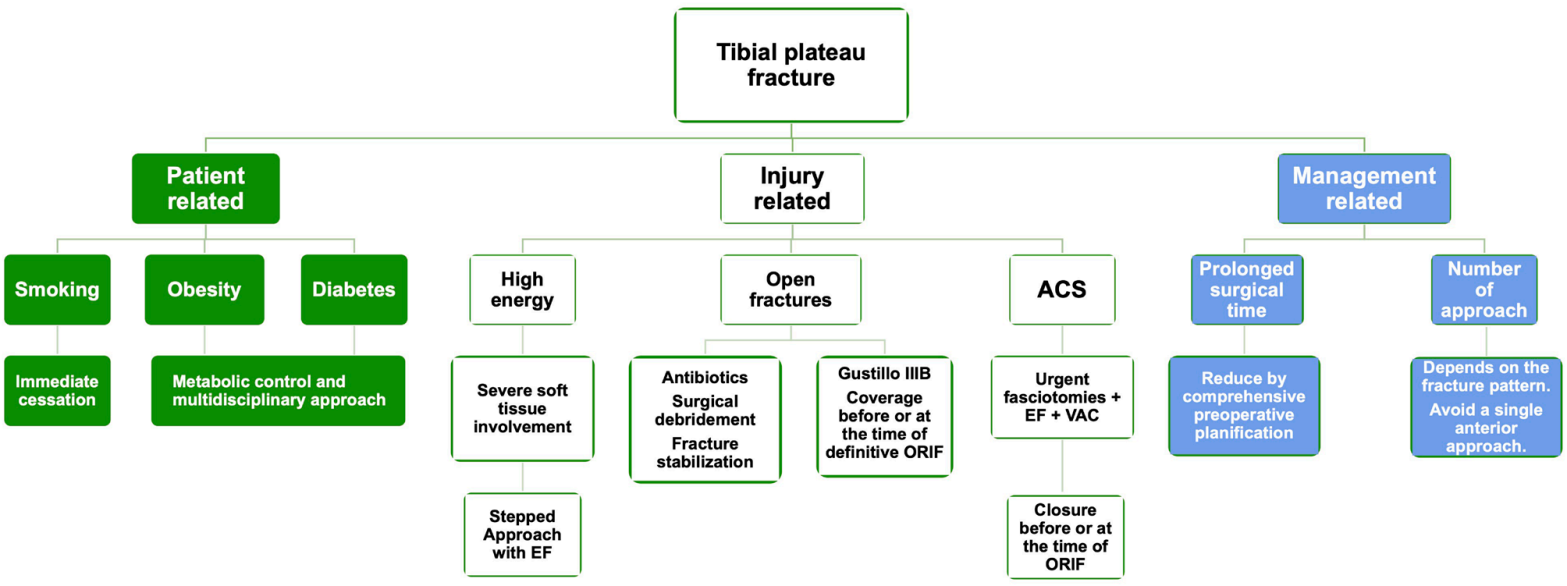
Algorithm for managing fracture-related infection risk factors in tibial plateau fractures. EF, external fixator; ORIF, open reduction and internal fixation; ACS, acute compartment syndrome; VAC, vacuum-assisted closure.

We consider that recognizing infection predictive factors in patients with tibial plateau fractures is crucial to achieve adequate FRI prevention. Based on the previously mentioned review, we are aware that there is ample literature supporting the relevance of the mentioned factors as predictors of infection. Despite there being more literature that dismisses their importance, we believe they deserve to be considered in all cases. To facilitate an appropriate approach, we find it useful to classify risk factors based on patient, injury, and management-related factors (Fig. 2).

Unfortunately, tibial plateau fractures are unexpected injuries, making it impossible to address patient-specific factors such as smoking, obesity, and diabetes in advance. However, we believe it is essential to educate the patient regarding the immediate cessation of smoking and optimizing their diet and metabolic control. In the case of patients with decompensated diabetes, we strive to request early evaluation and management by an endocrinologist or internist.

Regarding injury-related factors, we always carefully assess soft tissue conditions. In cases of moderate to severe involvement or if a high-energy mechanism is suspected, we recommend a stepped approach, initially using an external fixator. During its placement, we aim to position the pins in an area away from probable future surgical approaches. In the case of open fractures, proper treatment is achievable by considering early administration of antibiotics, appropriate surgical debridement, fracture stabilization, and early wound coverage. In the case of Gustilo IIIB open fractures, ensuring coverage at the time of definitive fixation is required. On the other hand, in the case of concomitant ACS, we perform coverage with vacuum-assisted closure (VAC) of the fasciotomies, surgical debridement at intervals of up to a maximum of 5 days, and aim to close the incisions either before or at the time of definitive osteosynthesis.

Regarding the definitive surgical management of the fracture, taking into account the aforementioned evidence, we believe that it is essential for the surgeon to try to reduce surgical time through various strategies, such as making a detailed and comprehensive preoperative assessment of the fracture, planning the reduction maneuvers in advance, and sharing the surgical plan with the other surgeons to work in a synchronized manner. As for the approach, it has been observed over the last two decades that the use of a single anterior incision to manage fractures of both plateaus has fallen out of favor due to high reported complication rates (4, 5, 59, 60). Due to this, we prefer to use approaches depending on the pattern of the fracture and the need for biomechanical fixation, and while the use of two or more approaches may imply a higher risk of infection, we believe that it is a well-justified strategy in fractures involving more than one column, especially including extended approaches.

Finally, regarding the use of antibiotics, in all cases we use antibiotic prophylaxis based on a single dose of preoperative cefazolin (1 or 2 g depending on the weight of the patient, less or greater than 80 kg, respectively) administered between 30 and 60 min before the initial incision. In cases of Schatzker V or VI fractures that require at least two approaches and involve an operating time >2 h, we apply 2 g of vancomycin powder distributed over the plates after performing the final cleaning with physiological saline solution and just before beginning the closure of the approaches (61). Similarly, we use vancomycin powder in cases of patients with ACS and open tibial plateau fractures, although we recognize that the existing evidence is not categorical for this conduct (62, 63, 64).

## ICMJE Conflict of Interest Statement

The authors declare that there is no conflict of interest that could be perceived as prejudicing the impartiality of the research reported.

## Funding Statement

This work did not receive any specific grant from any funding agency in the public, commercial, or not-for-profit sector.
